# Biosynthetic ε-poly-L-lysine for the treatment of extensively- and pan-drug-resistant *Pseudomonas aeruginosa*

**DOI:** 10.1038/s44259-025-00142-y

**Published:** 2025-09-09

**Authors:** Darren Shu Jeng Ting, Thet Tun Aung, Venkatesh Mayandi, Mercy Halleluyah Periayah, Eunice Tze Leng Goh, Mugil Muthu, Veluchamy Amutha Barathi, Jodhbir S. Mehta, Donald Tiang Hwee Tan, Rajamani Lakshminarayanan

**Affiliations:** 1 Academic Unit of Ophthalmology Department of Inflammation and Ageing, School of Immunity, Infection, and Inflammation, College of Medicine and Health, University of Birmingham, Birmingham, UK; 2 Birmingham and Midland Eye Centre, Sandwell and West Birmingham NHS Trust, Birmingham, UK; 3 Academic Ophthalmology, School of Medicine, University of Nottingham, Nottingham, UK; 4 Ophthalmology and Visual Sciences Academic Clinical Program, Duke-NUS Graduate Medical School, Singapore, Singapore; 5 Ocular Anti-Infectives & Inflammation Research Group, Singapore Eye Research Institute, Singapore, Singapore; 6 Translational Pre-Clinical Model Platform, Singapore Eye Research Institute, Singapore, Singapore; 7 Department of Ophthalmology, Yong Loo Lin School of Medicine, National University of Singapore, Singapore, Singapore; 8 Singapore National Eye Centre, Singapore, Singapore; 9 Eye and Cornea Surgeons, Eye and Retina Surgeons, Camden Medical Center, Singapore, Singapore; 10 Department of Pharmacy and Pharmaceutical Sciences, National University of Singapore, Singapore, Singapore

**Keywords:** Bacterial infection, Target validation, Clinical microbiology

## Abstract

*Pseudomonas aeruginosa* (PA) represents a major cause of antimicrobial resistance-related morbidity and mortality. The recent emergence of highly fatal infections, caused by carbapenem-resistant PA, has called for novel antimicrobial therapies and strategies. In this study, we highlight the therapeutic potential of ε-poly-L-lysine (εPL), an antimicrobial polymer for treating extensively-and pan-drug-resistant-PA. εPL displayed potent antimicrobial activity against all eight drug-resistant PA, including carbapenem- and polymyxin-resistant PA. It exhibited a low risk of AMR evolution, with no evidence of cross-resistance with polymyxin B (a last-line treatment for drug-resistant Gram-negative bacteria). We further demonstrated promising in vivo efficacy and safety of εPL against PA in a pre-clinical PA keratitis model, with comparable effects to topical levofloxacin (a gold standard treatment of infectious keratitis) in terms of clinical scoring, corneal health/thickness, and bacterial bioburden. In view of its broad-spectrum antimicrobial activity, low risk of AMR evolution and cross-resistance with existing last-line antibiotics, and general acceptance of safety when orally administered, εPL serves as a promising novel antimicrobial agent for further clinical development and translation to tackle antimicrobial resistance.

## Introduction

Antimicrobial resistance (AMR) has emerged as a major global health threat of the twenty-first century^1^. It is estimated that five million deaths were associated with bacterial AMR in 2019, and this number is likely to increase to 10 million deaths annually if AMR is not addressed by 2050. In addition, AMR poses significant impact on the healthcare resources (due to prolonged hospitalisation and treatment), work productivity and global economy, with a potential loss of hundreds of millions of dollars annually^1^.

*Pseudomonas aeruginosa* (PA), a Gram-negative opportunistic pathogen, represents a significant cause of bacterial AMR^2^. It is a member of the ESKAPEE pathogens, consisting of *Enterococcus faecium*, *Staphylococcus aureus*, *Klebsiella pneumoniae*, *Acinetobacter baumannii*, *Pseudomonas aeruginosa*, *Enterobacter spp*., and *Escherichia coli*, all of which are frequently associated with AMR-related morbidity and mortality^1^. PA is capable of causing a wide range of infections in humans, particularly pneumonia, urinary tract infection, septicaemia, burn infections, and infectious keratitis^3,4^. Its highly diverse AMR mechanisms have rendered many existing antibiotics ineffective, posing significant therapeutic challenges^2,5^. A recent study has highlighted the global emergence of carbapenem-resistant PA strains that cause serious infections that can be fatal^6^. In addition, a recent outbreak of an extensively carbapenem-resistant PA, linked to the use of artificial tears, was recently reported in the United States (https://www.cdc.gov/hai/outbreaks/crpa-artificial-tears.html). The bacterial isolates were resistant to all topical antibiotics used clinically. This unprecedented outbreak resulted in severe ocular, respiratory and systemic infections, leading to permanent visual loss and death. This outbreak was the first report on association between contamination of an artificial tear drop and bacterial keratitis (with subsequent systemic morbidity and mortality), highlighting an unmet need for new antimicrobial therapy for treating ocular and systemic multidrug-resistant (MDR)-PA. Furthermore, a prospective observational study, the Asia Cornea Society Infectious Keratitis Study (ACSIKS), involving Asian cohorts identified PA as the major bacterial pathogen of infectious keratitis, with ~20% of them demonstrating multidrug resistance^7^, highlighting the therapeutic challenges in infectious keratitis.

Cationic antimicrobial polymers have been shown promise as a novel group of antimicrobial agent in view of their potent antimicrobial activity and low toxicity to host cells^8^. Epsilon poly-L-lysine (εPL) is a natural biodegradable cationic antimicrobial polymer which was approved as a food preservative since early 2000s^9^. Similar to cationic antimicrobial peptides^10–12^, εPL exhibits strong antimicrobial and antibiofilm activities against a wide range of pathogens^9,13^, and primarily exerts its lethal effect through a membrane-permeabilising action^14,15^. Our previous studies^15,16^ have demonstrated broad-spectrum antimicrobial and antibiofilm activities of εPL against both antibiotic-susceptible and antibiotic-resistant bacteria (including ESKAPEE pathogens) and fungi, with minimal toxicity to host tissues. Extending from our previous positive experience, this study aimed to examine the therapeutic potential of εPL in treating MDR-PA, which may offer a potential novel solution for tackling AMR in eye infections.

## Results

### Antimicrobial susceptibility and resistance of *Pseudomonas aeruginosa* strains

Of all eight drug-resistant PA clinical isolates, two (PA-DX562 and PA-DX771) demonstrated pan-drug-resistant (PDR) to all 12 tested antibiotics, with the MICs being ≥8–64 times higher than the drug-susceptible quality control American Type Culture Collection (ATCC) PA strains (Table 1). Extreme drug resistance and multi-drug resistance were identified in five PA (PA-DX751, PA-DX737, PA-DX770, PA-DX783, and PA-DX744) and one PA (PA-DX768), respectively. εPL exhibited good antimicrobial efficacy against all eight PA clinical isolates (MIC = 8–32 µg/ml). Both ATCC PA strains (PA27853 and PA9027) were susceptible to all 12 antibiotics.

**Table 1:**
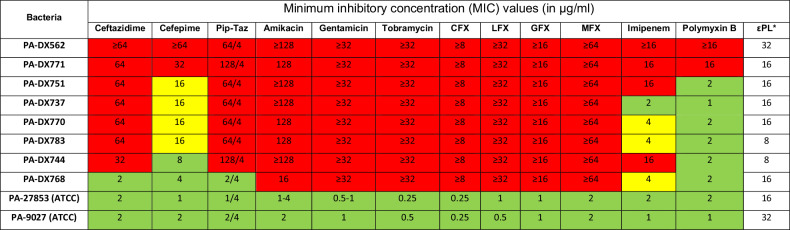
An antibiogram summarising the antibiotic susceptibility and resistance of eight drug-resistant and two drug-susceptible *Pseudomonas aeruginosa* (PA)

PA-DX refers to drug-resistant clinical strains of *Pseudomonas aeruginosa*; PA-27853 (ATCC) and PA-9027 (ATCC) are both laboratory drug-susceptible strain of *Pseudomonas aeruginosa* (often used as controls for drug testing). The antibiogram is encoded with red (resistance), yellow (intermediate), and green (susceptible) colours based on CLSI breakpoints. Intermediate susceptibility is considered as resistant in this study.

*PAPseudomonas aeruginosa*, *ATCC* American Type Culture Collection, *CFX* Ciprofloxacin, *LFX* Levofloxacin, *GFX* Gatifloxacin, *MFX* Moxifloxacin.

*As epsilon-polylysine (εPL) is not currently used in the clinic, therefore there is no breakpoint for this drug.

### Evolution of antimicrobial resistance

**Fig. 1:**
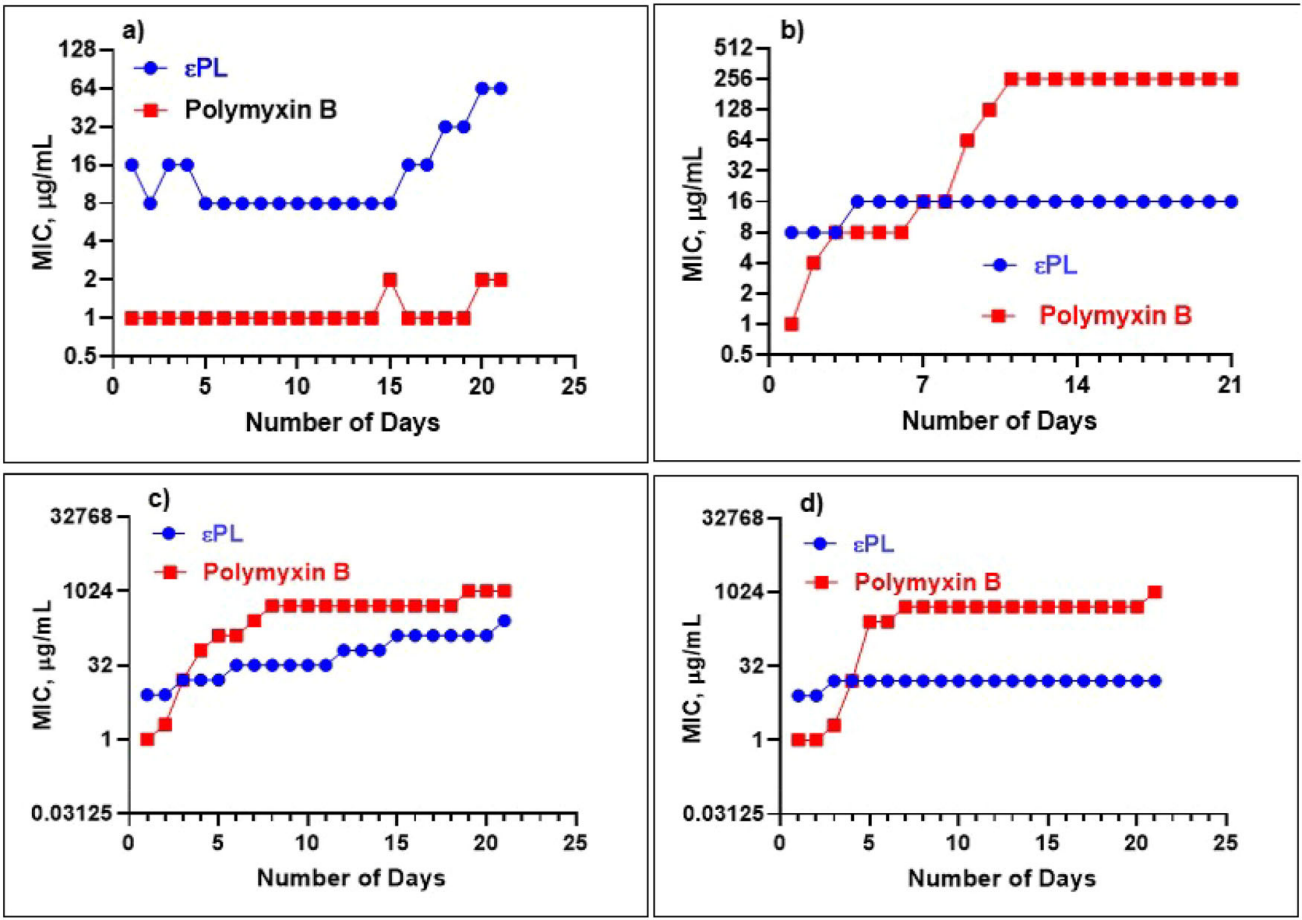
Serial passage resistance induction of εPL against antibiotic-susceptible and antibiotic-resistant Pseudomonas aeruginosa (PA) strains. MIC values for εPL and polymyxin B before and after each of 21 passages treated after sub-lethal exposure (½× MIC of εPL or polymyxin B). **a** ATCC PA9027; **b** ATCC PA 27853; **c** PA -DX783; and **d** PA -DX744. Except the PA9027 strain, the other three PA strains exhibited spontaneous and significant induction of AMR against polymyxin B, evidenced by a 128-fold increase in the MIC value (from 2 to 256 μg/ml) after 4–10 days of repeated sub-lethal exposure to polymyxin B treatment. Further increase in polymyxin B resistance was observed in XDR-PA after 18–20 days of sub-lethal exposure (MIC increased by 512-fold to 1024 μg/ml).

To determine the rate of potential resistance evolution, two ATCC strains (PA27853 and PA9027) and two clinical extreme drug-resistant (XDR)-PA strains (PA-DX783 and PA-DX744) were repeatedly exposed to sublethal concentration (½× MIC) of polymyxin B and εPL (Fig. 1a–d). Polymyxin B was chosen as the comparator antibiotic, as it is a last-line antibiotic that has the lowest resistance frequency amongst the other antibiotics. There was no significant risk of AMR observed against εPL in PA9027, PA27853 and PA-DX783 after repeated sub-lethal exposure, though AMR was observed in PA-DX744 against εPL after 21 days of treatment (a 32-fold increase). We further determined the susceptibility of polymyxin B-resistant terminal isolates, collected from day 21, for εPL. The results suggested that all the three polymyxin B-resistant strains were susceptible to εPL as the MIC values remained at 8–16 µg/ml against all the three polymyxin B-resistant strains.

### Time kill kinetics and mechanistic studies

**Fig. 2:**
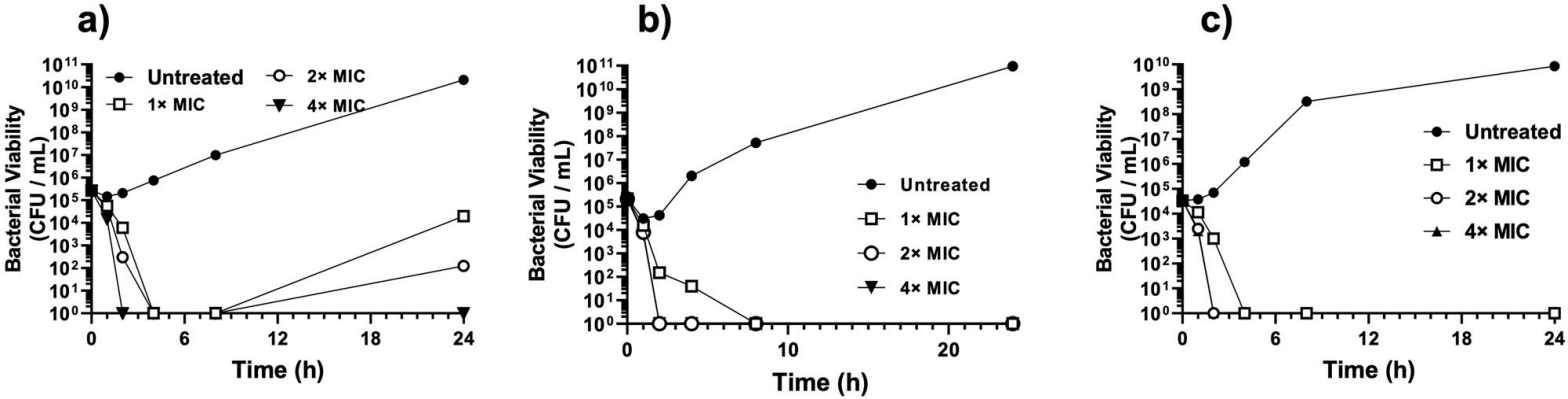
Rapid bactericidal activity of ePL against *P. aeruginosa* isolates. Time-dependent changes in bacterial viability after exposure to various concentrations of ePL is shown against ATCC and clinical isolates of *P. aeruginosa*. **a** PA 27853, **b** PA-DX783, and **c** PA-DX744. The concentration of ePL is expressed in terms of the respective MIC values against the bacterial isolates.

**Fig. 3:**
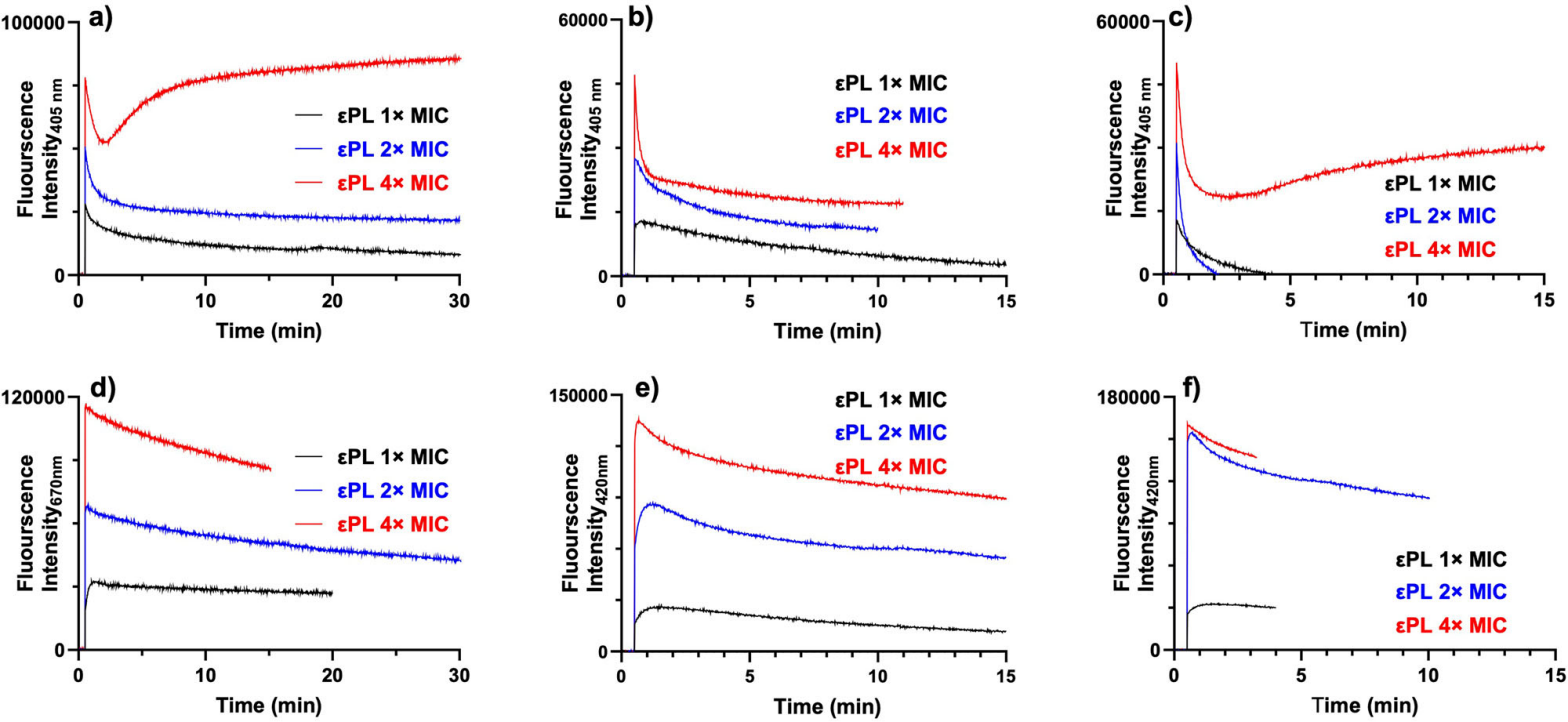
Outer membrane permeabilization and membrane depolarisation of *P. aeruginosa* by ePL. NPN assay showing the increase in the fluorescence intensity at 405 nm of the hydrophobic probe upon addition of ePL. **a** PA ATCC 27853; **b** PA-DX783; and **c** PA-DX744. DiSC_3_-5 assay showing the loss of membrane potential upon addition of increasing concentration of ePL. **d** PA ATCC 27853; **e** PA-DX783; and **f** PA-DX744.

Next, time kill kinetics experiment was performed after exposing antibiotic-susceptible and antibiotic-resistant bacterial cells to 1× - 4× MIC of εPL (Fig. 2). At 4× MIC values, complete bactericidal activity was observed within 2 h against the ATCC isolates whereas regrowth occurred after 8 h at low concentrations (1× or 2× MIC values). N-Phenyl-1-naphthylamine (NPN) assay demonstrated a concentration-dependent increase in outer membrane (OM) permeabilisation against all the three tested strains, suggesting disruption of OM and rapid permeabilisation of the hydrophobic probe (Fig. 3a–c). Similarly, 3,3’-Dipropylthiadicarbocyanine iodide (DiSC_3_-5) membrane potential sensitive assay confirmed polymer concentration-dependent rapid dissipation of cytoplasmic membrane potential upon addition of εPL to the intact bacterial cells (Fig. 3d–f).

### In vivo antimicrobial efficacy and safety of εPL in pre-clinical mice and rabbit studies

**Fig. 4:**
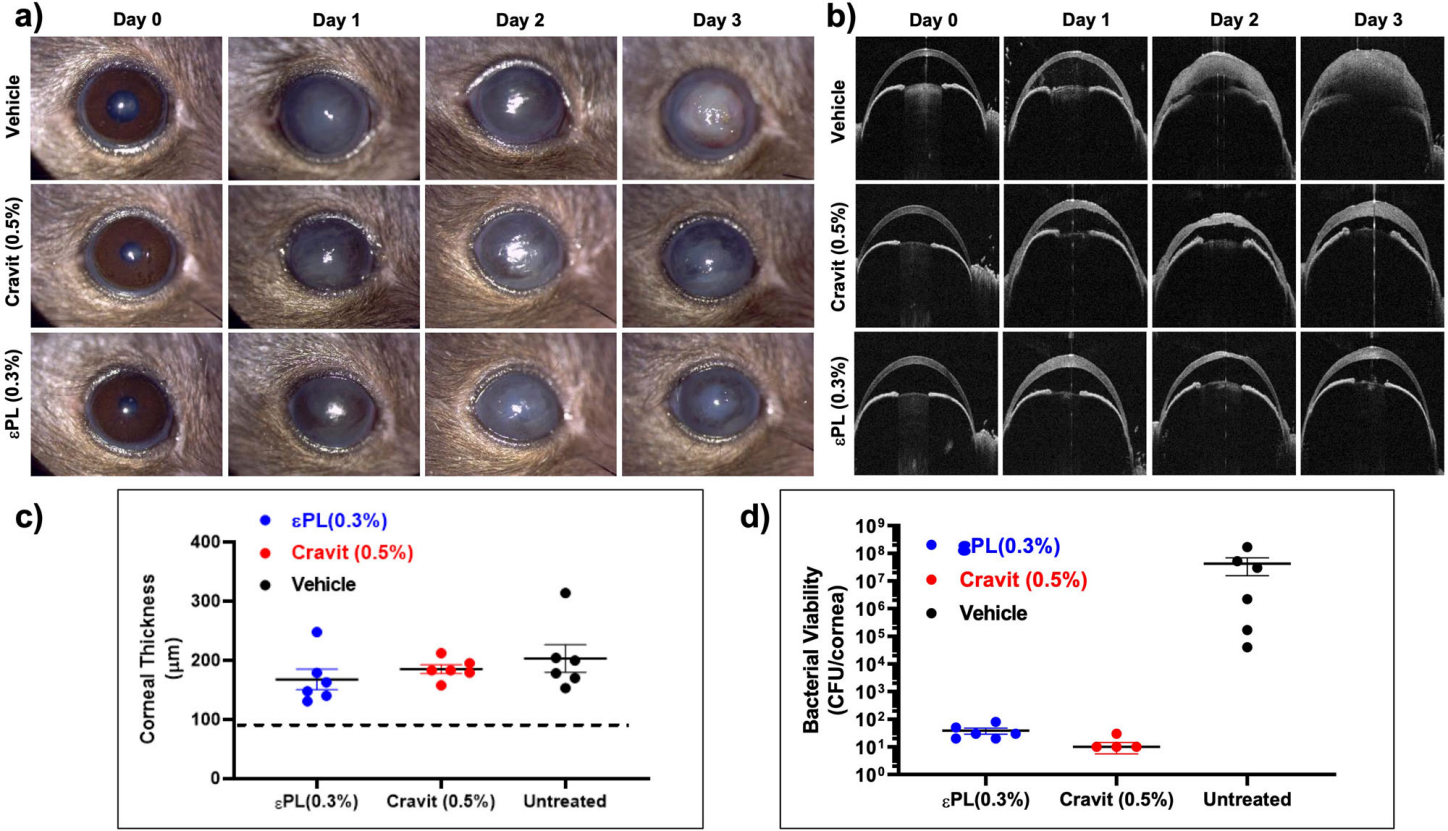
Efficacy and safety of εPL in *Pseudomonas aeruginosa* keratitis mice model. **a** Slit lamp images showing the progression of infection after treatment with various treatment groups, including εPL 0.3%, levofloxacin (Cravit) 0.5%, and PBS (vehicle, untreated) groups. **b** AS-OCT images showing the changes in corneal thickness after infection in three treatment groups over 3 days. Infected eyes that received εPL or levofloxacin showed substantially less inflammation than those that received vehicle alone. **c** Corneal thickness after treatment with various groups at 3 days post-infection. Note that εPL treatment (except for one eye) resulted in less corneal oedema/thickening when compared to eyes that received vehicle or levofloxacin. **d** Bacterial burden in the cornea after 3 days of treatment. The mean bacterial bioburden (±SD) for each treatment group is presented. εPL- and levofloxacin-treated groups showed significant reduction in the bacterial bioburden when compared to the PBS-treated group (*p* = 0.004 for both comparisons), but no difference was noted between εPL- and levofloxacin-treated groups (*p* = 0.085; Mann–Whitney *U* test). A standardised ocular clinical scoring system was used^30^: **a** 0: Clear cornea or minimal opacity, partially covering the pupil; **b** +1: Mild opacity, partially/fully covering the pupil; **c** +2: Dense opacity, partially covering the pupil; **d** +3: Dense opacity, fully covering the pupil; and **e** +4: Corneal perforation or phthisis. After 48 h post treatment with various groups, 2/6 (33%) mice in the εPL/levofloxacin treated eyes had grade 3 severity score when compared to 5/6 (83.3%) for the vehicle-treated group.

The pre-clinical mice study demonstrated the efficacy and safety of εPL in treating PA keratitis, based on the improvement in clinical ocular severity scoring, bacterial bioburden, and corneal thickness. The clinical severity score was lower in the εPL-treated group (2.16 ± 0.75) and the levofloxacin-treated group (1.83 ± 0.98), when compared to the PBS-treated groups (2.66 ± 0.82; *p* = 0.24; Mann–Whitney *U* test) (Fig. 4a). When compared to the untreated infected control (6.6 ± 1.3 logCFU), both εPL (1.5 ± 0.2 logCFU) and levofloxacin treatment (1.1 ± 0.2 logCFU) resulted in a significant decrease in bacterial burden (*p* = 0.004; Mann–Whitney *U* test; Fig. 4d). Anterior segment optical coherence tomography (AS-OCT) images showed less hyper-reflective material and corneal oedema (a proxy for corneal inflammation) in the εPL-treated groups in comparison to PBS- or levofloxacin-treated groups (Fig. 4b). When compared to untreated control (203.2 ± 57.3 μm), the eyes that received εPL treatment (167.9 ± 42.6 μm; *p* = 0.15; Mann–Whitney *U* test) and levofloxacin-treated (185.1 ± 18.0 μm; *p* = 1.0; Mann–Whitney *U* test) had a lower average central corneal thickness (CCT), highlighting less corneal oedema (though non-statistically significant, Fig. 4c). In addition, AS-OCT images indicated that 4/6 (66.7%) eyes in the levofloxacin-treated/control eyes had substantial anterior chamber inflammation whereas 2/6 (33.3%) eyes in the εPL-treated group displayed signs of anterior chamber inflammation (Supplementary Fig. S1).

**Fig. 5:**
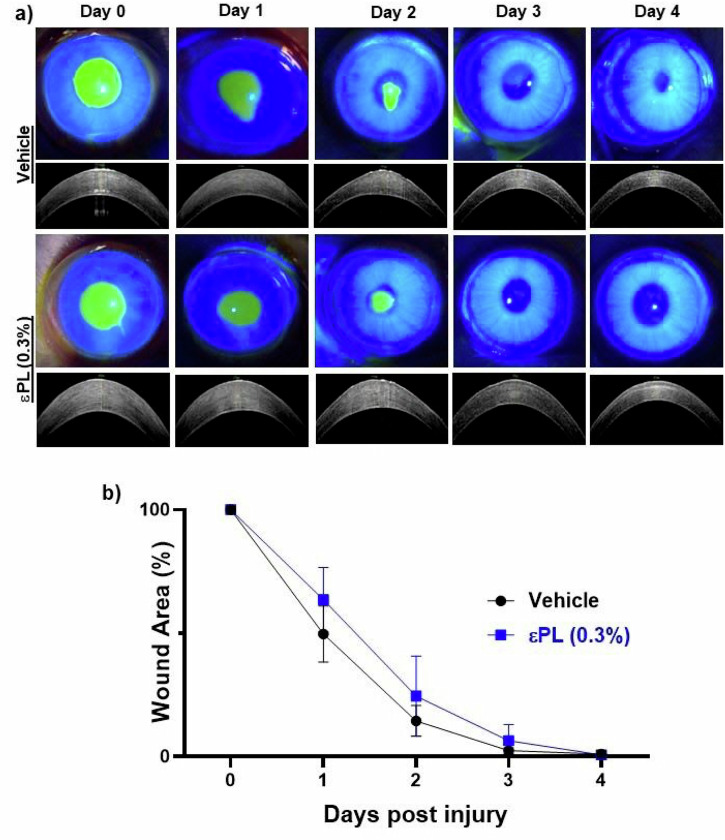
In vivo ocular safety of εPL in a rabbit cornea wound healing model. **a** Representative fluorescent slit lamp (top panel) and anterior segment optical coherence tomography (AS-OCT) images (bottom panel) showing the progression of fluorescein staining and corneal thickness, respectively, of injured cornea after topical instillation of vehicle alone or 0.3% εPL solution (w/v). Rabbits’ corneas were treated with respective treatment four times a day for 96 h. AS-OCT images indicated that after an initial increase in corneal thickness immediately after injury, both vehicle- and εPL-treated corneas approached the baseline values. **b** Changes in wound area upon treatment with vehicle or εPL were monitored daily over 96 h (*n* = 6 eyes/group). For all the time points *p* > 0.05, by Mann–Whitney test.

**Fig. 6:**
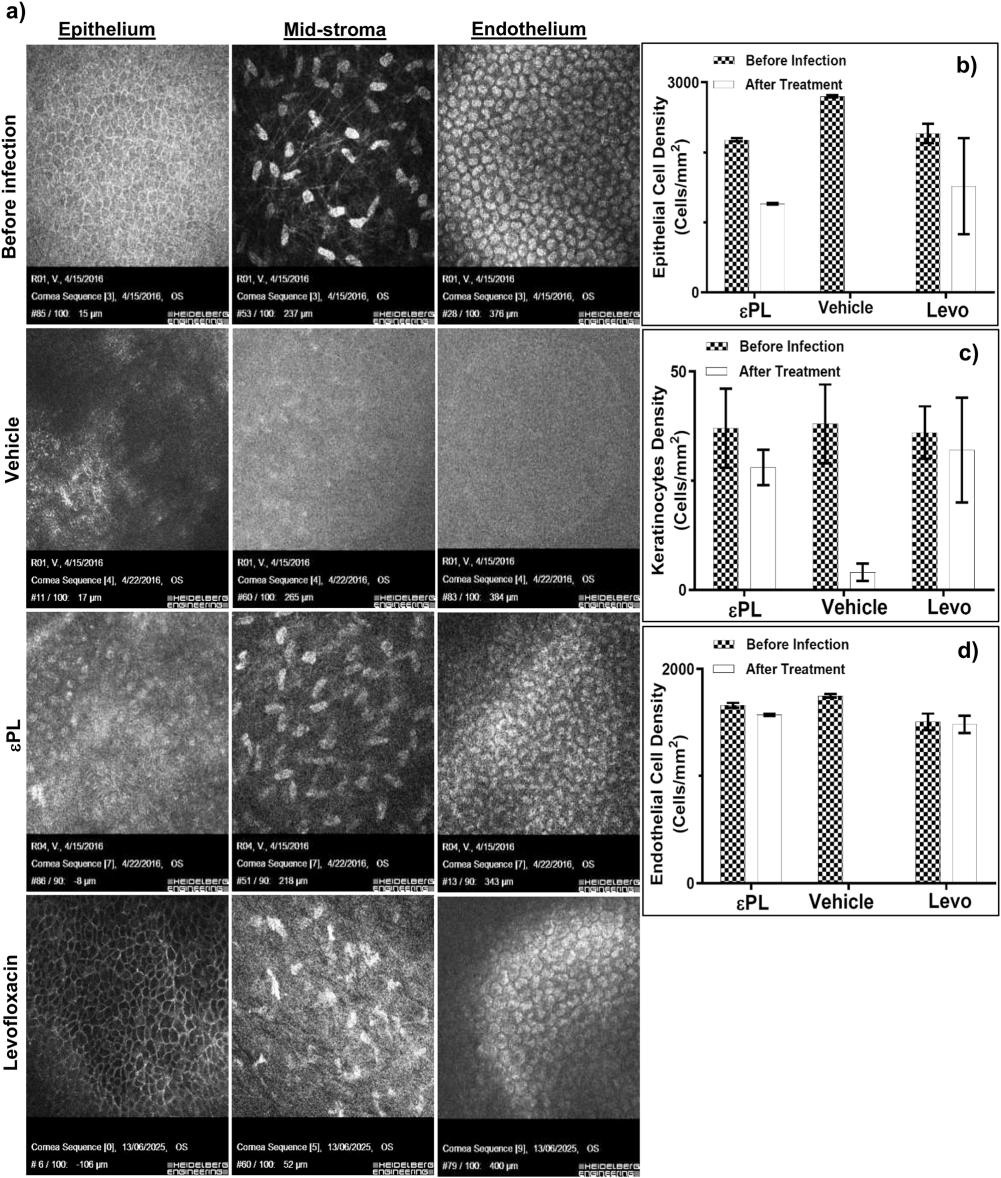
Corneal microarchitecture of vehicle- and εPL-treated rabbit corneas. **a** In vivo confocal microscopic images showing the features of various layers of the cornea before infection and 72 h after treatment with vehicle and εPL. Bar graphs showing the corneal epithelial (**b**), keratocytes (**c**) and endothelial (**d**) cellular density before infections and after treatment with εPL or vehicle (n = 4 eyes/group). Prior to infection, three cellular layers of the cornea, including epithelium, mid-stroma and endothelium, were visible (**a**, top panel). At 3 days post treatment, the imaging was difficult to achieve owing to light attenuation in the vehicle-treated group (**a**, middle panel). However, in the εPL-treated cornea, substantial improvement in various layers could be seen, indicating clearance of the bacterial infection (**a**, bottom panel). **b–d** Quantification of cellular densities of the three layers confirmed significant recovery of epithelial and keratocytes cell densities without apparent change in the endothelial cell density for the εPL-treated cornea. Comparative analysis of the corneal microarchitecture and cellular density between the vehicle- and εPL-treated corneas showed significant difference between the two groups at 3 days post treatment: **b** epithelial cell density [pre-infection: εPL (2179 ± 24 cells/mm2 vs Levo (2270 ± 137 cells/mm2) vs. PBS (2802 ± 16 cells/mm2); post-infection: εPL (1267 ± 12 cells/mm2) vs Levo (1518 ± 686 cells/mm2) vs PBS (0 cells/mm2)]; **c** keratocytes density [pre-infection: εPL (37 ± 9 cells/mm2 vs Levo (36.3 ± 6 cells/mm2) vs. PBS (38 ± 9 cells/mm2); post-infection: εPL (28 ± 4 cells/mm2) Levo (32±12 cells/mm2) vs PBS (4 ± 2 cells/mm2); **d** endothelial cell density [pre-infection: εPL (1662 ± 21 cells/mm2) vs Levo (1505 ± 78 cells/mm2) vs. PBS (1749 ± 19 cells/mm2); post-infection: εPL (1570 ± 10 cells/mm2) vs Levo (1483 ± 81 cells/mm2) vs PBS (0 cells/mm2)].

Ocular toxicity of the topical εPL (0.3% w/v in PBS) formulation was carried out in a rabbit model of corneal injury. Analysis of the fluorescent slit lamp images demonstrated that there was no significant difference in the re-epithelialisation rate between vehicle and εPL treatment (Fig. 5a, b). AS-OCT images further indicated that εPL treatment did not cause any adverse effect to the cornea (e.g. corneal melt/oedema) after repeated topical instillation (Fig. 5a). To further confirm and characterise the antimicrobial activity of εPL, we performed IVCM in a rabbit PA keratitis model. A comparative analysis of the corneal microarchitecture and cellular density among the vehicle-, εPL-, and levofloxacin-treated corneas further established the potent antimicrobial properties and safety of εPL and comparator antibiotic (Fig. 6). The analysis showed significant recovery of epithelial and keratocytes cell densities without apparent change in the endothelial cell density for the εPL- or levofloxacin-treated cornea (Fig. 6). Taken together these results established the potent antimicrobial properties and safety of εPL for the treatment of PA keratitis.

## Discussion

The rapid emergence of bacterial AMR in the past decade has significantly challenged the antimicrobial therapeutic armamentarium. This crisis is further compounded by the dwindling interest in new antibiotic drug discovery and development, due to the lack of financial incentives and pharmaceutical investment^1^. In this work, we highlighted the potential of εPL in treating XDR-PA and PDR-PA, with a low risk of AMR evolution. In addition, there was no evidence of cross-resistance between εPL and polymyxin B. The in vivo efficacy and safety of topical εPL was further substantiated in a pre-clinical antibiotic-susceptible PA keratitis model, with comparable effects to topical levofloxacin in terms of clinical score and bacterial bioburden (one of the gold standard treatment for treating PA keratitis in the clinic). εPL treatment resulted in less oedematous cornea than those that received levofloxacin or PBS treatment, suggesting beneficial effect of the polymer in comparison to fluoroquinolone antibiotics.

PA is an opportunistic Gram-negative pathogen equipped with highly diverse mechanisms for AMR, including intrinsic, adaptive and acquired resistance, making it an extremely difficult-to-treat organism^2^. In 2024, carbapenem-resistant PA was listed as a high priority pathogen by the WHO in view of its capability to cause fatal infections such as pneumonia and septicaemia^1,6^. PA has been shown to be a leading cause of pneumonia (particularly in patients with cystic fibrosis)^17^ and infectious keratitis (especially in contact lens wearers due to its biofilm-forming ability)^18,19^. In the ACSIKS, PA was identified as the most common organism, accounting for ~10% of the total organisms isolated^18^. In addition, 80% of MDR-PA were from India^7^. This was consistent with previous reports which have demonstrated an increase in drug-resistant PA keratitis in several countries, particularly China and India^20^. These drug-resistant PA have been shown to result in poorer clinical outcome and delayed corneal wound healing^21^.

In our study, all eight PA were resistant to fluoroquinolone and aminoglycosides, both commonly used antibiotics for PA-related infectious keratitis. Moreover, 87.5% of the included PA demonstrated resistance to imipenem, and 25% of them exhibited PDR against all types of antibiotics, including fluoroquinolone, aminoglycosides, cephalosporin, polymyxin, and carbapenem. All XDR- and PDR-PA included in this study were obtained from clinical specimens of patients with severe infectious keratitis at the Indian site of ACSIKS. Although the impact of infectious keratitis is typically limited to the eye, the recent outbreak of VIM-GES-CRPA, which was linked to the use of contaminated artificial tears manufactured in India and resulted in significant ocular infection and/or death, has raised serious concerns about the rise in XDR-PA and the potential of ocular-to-systemic transmission of highly fatal, antibiotic-resistant infections.

Encouragingly, εPL exhibited good and comparable efficacy against all eight MDR-, XDR- and PDR-PA (MIC = 8–32 µg/ml), with no evidence of cross-resistance with all 12 tested antibiotics. The lack of cross-resistance is likely attributed to the unique and rapid antimicrobial action of εPL (via membrane-permeabilising mechanisms)^14,15^ as opposed to the mechanisms of conventional antibiotics, which primarily target certain outer membrane or intracellular components. The rapid antimicrobial action and the relatively higher cost of microbial fitness (to modify the overall bacterial membrane instead of certain intracellular targets) to resist εPL are likely accountable for the low risk of εPL resistance. On the other hand, polymyxins [including polymyxin B and E (also known as colistin)] are cationic lipopeptide antibiotics, which exert their bactericidal effect through interaction and disruption of the anionic bacterial membrane, similar to εPL. However, polymyxin resistance has been reported, which is primarily attributed to the modification of the lipopolysaccharides in the OM of Gram-negative bacteria^1^.

In view of the similar mechanism of action of polymyxin B and εPL, we selected two XDR-PA (that were susceptible to both polymyxin B and εPL) and two antibiotic-susceptible ATCC strains (PA9027 and PA27853) to examine the risk of AMR evolution and cross-resistance. It is striking to observe a significant increase in polymyxin resistance in XDR-PA and PA27853 after several consecutive days of sub-lethal treatment of polymyxin B, whereas εPL resistance was observed in only one XDR-PA (and to a significantly lesser extent than polymyxin resistance). Interestingly, we did not observe any cross-resistance between polymyxin B and εPL, evidenced by the good susceptibility of engineered polymyxin B-resistant XDR-PA (after 21 sub-lethal treatment passages) to εPL (MIC remained at 8–16 µg/ml). This suggests that εPL may be utilised to treat polymyxin-resistant bacteria as the mechanism of AMR between the two drugs is likely to be different. Further bacterial genomic studies may provide further insights into the molecular mechanism of AMR for εPL.

Time-kill kinetics studies confirmed that both the antibiotic-resistant isolates were hyper sensitive to the antimicrobial action of εPL when compared to ATCC isolates, as complete bactericidal effect was observed at 2× MIC values of the polymer whereas similar effect was observed at 4× MIC against the susceptible isolates. NPN and fluorescence studies confirmed rapid OM permeabilization and disruption of cytoplasmic membrane potential upon addition of polymer to bacterial cells. Taken together, these results confirm the membrane perturbing action of εPL against the pathogenic bacteria. It is likely that the lack of evolution of antimicrobial resistance for εPL is attributed to the membrane active mechanism of action and the concomitant heightened fitness cost associated with the reconstruction of the entire cytoplasmic membrane.

In our pre-clinical mice study, we observed that topical εPL, applied four times daily over 3 days, were able to achieve significant eradication of the PA infection (~99.999% reduction in the bioburden) with relative preservation of the corneal transparency when compared to the PBS-treated group. Clinically, patients affected by infectious keratitis are often treated with hourly antibiotics for the first few days before gradual tapering of the treatment. Therefore, it is likely that intensive topical application of εPL (hourly over several days) could help achieve complete eradication and resolution of the infection. Repeated topical instillation of therapeutic dose of the polymer did not alter the wound healing of injured rabbit cornea, confirming the lack of ocular toxicity of εPL. AS-OCT demonstrated an initial increase in corneal thickness in both εPL and PBS groups immediately post-corneal injury, followed by a return toward baseline values over 96 h. The oedematous cornea occurring after the corneal epithelial injury is due to the damage and breach of the tight junctions of corneal epithelial basement membrane, resulting in compromised cellular function and inflammation, which results in imbibition/influx of water into the corneal stroma. As the epithelial injury heals, the excess fluid is resorbed and pumped out from the cornea into the anterior chamber through the corneal endothelial pump action, leading to normalisation of corneal thickness.

IVCM characterisation of the PA-infected rabbit cornea treated with εPL further established antimicrobial potency of the polymer. Ophthalmic drugs are frequently formulated to decrease the number of applications by enhancing the residence time so as not be washed away by tears. Using such formulations the frequency of dosing could be reduced. It could also help efficacy as tissue penetration could be increased. Although PA9027 was used in our pre-clinical study (instead of an XDR- or PDR-PA), we expect to observe a similar in vivo efficacy of εPL for these antibiotic-resistant organisms as the MIC of PA9027, XDR-PA and PDR-PA for εPL was similar (≤32 µg/ml). The reason for not using an XDR-PA or PDR-PA in our pre-clinical murine model was due to the restriction of our institutional policy and regulation (in view of the high risk of biosafety, AMR transmission and health hazard).

Among several described emerging approaches, εPL has a multitude of advantages for further clinical translation as a novel therapy for tackling AMR. Firstly, since it is generally recognised as safe (GRAS) by the FDA [(up to 50 mg/kg (ppm)] as a food preservative, the probability of good safety of using εPL as eye drops is high, though ocular irritation tests of ophthalmic formulations remain to be investigated. Second, it has potent and broad-spectrum antimicrobial activities against a range of organisms, including XDR-PA and PDR-PA, as shown in our current and previous studies^15^. Third, it has strong activity against PA biofilm^15^, which is a well-recognised mechanism by which PA (including carbapenem-resistant PA) enhances its virulence and resistance against antibiotics^22,23^. εPL has also displayed antifungal and anti-amoebic activities, highlighting its broad-spectrum activities against various ocular pathogens^24,25^. Its absence of cross-resistance with polymyxin and carbapenem may serve as a potentially novel rescue therapy for XDR- and PDR-PA in the clinic. εPL can also be developed as an active constituent in an antimicrobial dressing (for treating acute and chronic wounds)^16^, as an antimicrobial hydrogel^26,27^, and as an antimicrobial coating for medical devices and implants^27^. Interestingly, AS-OCT images suggested that εPL decreased the anterior chamber inflammation in comparison to levofloxacin demonstrating its ability to control infections and bacteria-induced inflammation. Lastly, recent advancement in the biomanufacturing process and reduced cost of εPL (a major factor that limits the therapeutic utility of membrane-active antimicrobial peptides) has rendered it an attractive antimicrobial agent for further clinical translation for various ocular infections.

In conclusion, this study highlights the therapeutic potential of εPL in treating MDR-, XDR- and PDR-PA with minimal risk of AMR evolution. Further pre-clinical studies utilising MDR clinical isolates in various infectious keratitis and systemic infection models would be invaluable. In addition, pharmacokinetics of εPL in ocular tissues will be conducted prior to human clinical trials. These studies will also serve as an important stepping stone for clinical translation for treating MDR ocular and/or systemic infections in human.

## Methods

### Study design

This study was conducted using a combination of in vitro and in vivo experiments. All experiments were conducted in at least two biological replicates and repeated in three independent experiments. Relevant positive and vehicle/negative controls were used in all experiments.

### Materials

Twelve medically important/last-line antibiotics, including third- and fourth-generation cephalosporins (ceftazidime and cefepime), piperacillin-tazobactam, aminoglycosides (amikacin, gentamicin and tobramycin), second-, third- and fourth-generation fluoroquinolones (ciprofloxacin, levofloxacin, moxifloxacin, and gatifloxacin), imipenem, and polymyxin B, were tested in this study. They were all purchased from Sigma-Aldrich Ltd, Singapore. εPL (*M*_*w*_, ∼4000) was purchased from Hefei TNJ Chemical Industry Co. Ltd., China. Two American Type Culture Collections (ATCC) quality control reference strains of PA, including ATCC PA27853 and ATCC PA9027, were purchased from Bio-rev (Chemopharm Group), Singapore.

### In vitro antimicrobial efficacy

In vitro antimicrobial efficacy of antibiotics and εPL was determined against two ATCC strains (PA27853 and PA9027) and eight MDR-PA that were clinically isolated from patients with infectious keratitis as part of the ACSIKS^7,18^. PA-DX562 was isolated from a patient at All India Institute of Medical Sciences, New Delhi, India while the other drug-resistant PA were isolated from seven different patients at LV Prasad Eye Institute, Hyderabad, India^7^. Established minimum inhibitory concentration (MIC) assays with the broth microdilution method were performed, and antibacterial susceptibility breakpoints were determined based on the Clinical and Laboratory Standards Institute (CLSI) guideline (Supplementary Table 1)^28^. MDR was defined as non-susceptibility to ≥1 agent in three or more antimicrobial categories, extensively drug-resistant (XDR) was defined as non-susceptibility to ≥1 agent in all but two or fewer antimicrobial categories, and pan-drug-resistant (PDR) was defined as non-susceptibility to all agents in all antimicrobial categories^29^. Intermediate susceptibility was considered as resistance in this study.

### Serial passage antimicrobial resistance study

Serial passage AMR assays were performed to evaluate the evolution of AMR of two PA ATCC reference strains (PA9027 and PA27853) and two XDR-PA (PA-DX783 and PA-DX744) against polymyxin B (a last-line treatment for MDR Gram-negative bacteria) and εPL over 21 consecutive passages (days)^30,31^. After determining the MIC level of each treatment at baseline (day 1), treated bacterial suspensions were obtained from the 0.5x MIC well of each treatment and were adjusted in MHB to achieve a final bacterial concentration of ~1 × 10^6^ CFU/ml. Subsequently, 100 μl of 1 × 10^6^ CFU/ml of the bacteria was added to the corresponding wells containing serially diluted treatment, and the MIC was determined after 24 h. Development of mild and significant AMR was defined as a ≥4-fold and ≥16-fold increase in the MIC level compared to the baseline, respectively.

### Time kill kinetics assay

The concentration-dependent changes in the kinetics of bactericidal activity of εPL was confirmed by time-kill kinetics assay. Bacterial isolates (10^5^–10^6^ CFU/ml in MHB) were exposed to εPL with two-fold increasing polymer concentrations in a glass test tube and incubated at 37 °C under continuous agitation. Ten μL aliquot of the samples in duplicates were withdrawn at various time intervals (1, 2, 4, 8 and 24 h) and the log_10_-fold dilutions were plated onto a tryptic soy agar (TSA) plate for CFU enumeration. Isolates without the polymer served as control.

### Outer membrane permeability assay

Bacteria cells were grown overnight in MHB and washed with 5 mM HEPES buffer containing 5 mM glucose. The cells concentration was adjusted to OD 0.4 and incubated with 10 μM of N-phenyl-1-naphthylamine (NPN). About 600 μL of the dye-loaded cell suspension was transferred to a quartz cuvette. The fluorescence intensity was monitored using a Quanta Master spectrofluorometer (Photon Technology International, NJ, USA) at excitation 355 nm and emission 405 nm. Once a stable baseline was obtained the polymer was added and the fluorescence emission intensity at 405 was recorded. The final εPL concentrations tested were 1×, 2×, and 4× MICs.

### Membrane depolarisation assay

The overnight grown bacterial culture was adjusted to OD_600_ ~ 0.4 and incubated with 10 μM of membrane potential sensitive probe, 3′,3′-dipropylthiadicarbocyanine (DiSC_3_-5), for 1 h in dark at room temperature. About 600 μL of the dye-loaded cell suspension was transferred to a quartz cuvette and εPL was added after stable fluorescence signal was obtained. The final εPL concentrations tested were 1×, 2×, and 4× MICs. The fluorescence intensity was monitored using a Quanta Master spectrofluorimeter (Photon Technology International, NJ, USA) at excitation 622 nm and emission 670 nm.

### Animal ethics and study conduct

The efficacy and safety of εPL were further validated using established pre-clinical mice and rabbit PA keratitis models. All animal studies were conducted at the Singapore Eye Research Institute, Singapore, and were approved by the Institutional Animal Care and Use Committee (IACUC) and the Institutional Biosafety Committee (IBC) of SingHealth, Singapore (Ref: 2019/SHS/1491). The animals were maintained and treated in compliance with the Guide for the Care and Use of Laboratory Animals (National Research Council, Singapore) and the ARVO statement for the Use of Animals in Ophthalmic and Vision Research. The in vivo experiments complied with the ARRIVE guidelines (https://arriveguidelines.org) and were carried out in accordance with the UK Animals (Scientific Procedures) Act 1986. All assessors/graders were masked from the treatment allocation to avoid assessor/observer bias.

### Efficacy and safety of εPL in PA keratitis mice study

The in vivo antimicrobial efficacy and safety of εPL were examined using an established pre-clinical murine bacterial keratitis model^30,31^. All mice (*n* = 4–6 mice/group) were randomly allocated to each treatment group, namely phosphate-buffered saline (PBS; negative control), levofloxacin 0.5% (Cravit, Santen Pharmaceutical Asia Pte Ltd., Singapore), a commonly used antibiotic for PA keratitis in the clinic^32^, and εPL (0.3% w/v). After the administration of general anaesthesia [with intraperitoneal injections of xylazine (10 mg/kg) and ketamine (80 mg/kg) (Ilium Troy, Australia)] and topical anaesthesia (with proxymetacaine hydrochloride 0.5%) (Bausch & Lomb, UK), the central 2 mm corneal epithelium was gently removed with sterile mini-blades (Beaver, MA, USA). Ten μl of ~ 5 × 10^6^ CFU/ml of PA9027 was applied topically onto the cornea and the lid was held shut for 1 min. At 24 h post-infection, 10 μl of respective treatment was applied directly onto the infected corneas four times daily (3 h apart) for 3 days (a total of 12 doses). The eyes were monitored daily with slit-lamp biomicroscopic photography and anterior segment optical coherence tomography (AS-OCT). A standardised ocular clinical scoring system was used^30^: AS-OCT (RTVue, Optovue, Fremont, CA) was used to measure the corneal thickness perpendicular to the anterior corneal surface at baseline and post-infection. Corneal tissue loss or oedema, defined as the difference in central corneal thickness (CCT) between baseline and day 3 post-infection, was calculated^15^. Mean difference between groups were analysed using *T*-test or Mann–Whitney *U* test where appropriate.

At the end of day 3, the mice were sacrificed according to the method of humane killing set out in Animals (Scientific Procedures) Act 1986 Schedule 1 using overdose of general anaesthesia via intraperitoneal route. The whole corneas were dissected and homogenised in 1 ml of sterile PBS using sterile 2 mm diameter glass beads. The homogenised infected corneal tissue suspension was serially diluted in 1:10 and plated on TSA plates in triplicates for enumeration of CFU after 24 h incubation at 37 °C. The main outcome measures were the bacterial load at 3-day post-infection (expressed as logCFU/ml, equivalent to logCFU/cornea), ocular clinical scoring, and corneal thickness. The mean difference between two groups was analysed using Mann–Whitney *U* test due to non-normal distribution of the data.

### Safety of εPL in corneal wound healing rabbit study

Six New Zealand white rabbits, aged 2–4 months and weighed 3–3.5 kg, were used for the study and were divided into two groups. A 6-mm diameter circular region of the corneal surface was de-epithelialised with a sterile mini blade (BD Beaver, MA, USA) after anaesthetising the rabbits. The two groups of rabbits received 50 μl topical instillation of 0.3% εPL (w/v in PBS, pH 7.0) or PBS at 4 times/day until complete wound closure. The corneal epithelial wound healing was visualised by the addition of a drop of 0.5% w/v sodium fluorescein (Bausch & Lomb), which could help reveal any epithelial defects upon illumination with a cobalt blue filter. The corneas were photographed immediately after wounding as well as at 1, 2, 3, and 4 days post-injury. The area of the epithelial defects was then estimated by ImageJ software. AS-OCT scans were taken before and after infection as well as during the course of the treatment. Difference in the wound size between groups was analysed using one-way ANOVA with Dunnett’s post hoc test (PBS as the control group).

### *Pseudomonas aeruginosa* keratitis rabbit study

New Zealand white rabbits (*n* = 4), aged 1–2 months and weighed 2–2.5 kg, were used for this study. The rabbits were anaesthetised in a similar manner as above, and the corneal surface was de-epithelialised with a sterile mini blade (BD Beaver, MA, USA). Corneal infection was induced by applying 50 μl of PA9027 strains (5 × 10^6^ CFU/ml) to the scarified cornea. After 24 h post-infection, 50 μl of 0.3% epsilon poly-L-lysine (εPL) or PBS were applied topically to the infected eyes at 4 times/day. Slit-lamp biomicroscopy/photography and AS-OCT were performed daily, and IVCM imaging (HRT3 Rostock module; Heidelberg Engineering GmbH, Heidelberg, Germany) were taken at baseline and 96 h post-infection and reported. Similar analyses of the main outcome measures were performed as the PA keratitis mice study.

## Supplementary information

Supplementary Table and Figure.

## Data Availability

The authors confirm that the data supporting the findings of this study are available within the article and its Supplementary Materials.
