# Supplementary material for: Biosynthetic ε-poly-L-lysine for the treatment of extensively- and pan-drug-resistant Pseudomonas aeruginosa
Source: NPJ Antimicrob Resist. 2025 Sep 9;3:77. doi: 10.1038/s44259-025-00142-y (PMC12420815; doi:10.1038/s44259-025-00142-y)

**Supplementary Table 1.** Clinical susceptibility breakpoints of various antibiotics against *Pseudomonas aeruginosa* based on the CLSI guideline.

| **Antibiotics** | **Susceptible (µg/ml)** | **Resistant (µg/ml)** |
| --- | --- | --- |
| Cephalosporins |  |  |
| Ceftazidime (3^rd^ generation) | ≤8 | ≥32 |
| Cefepime (4^th^ generation) | ≤8 | ≥32 |
| Penicillin-beta lactamase inhibitor |  |  |
| Piperacillin-tazobactam* | ≤16 | ≥64 |
| Aminoglycosides |  |  |
| Amikacin | ≤4 | ≥16 |
| Gentamicin | ≤2 | ≥8 |
| Tobramycin | ≤1 | ≥4 |
| Fluoroquinolones |  |  |
| Ciprofloxacin (2^nd^ generation) | ≤0.5 | ≥2 |
| Levofloxacin (3^rd^ generation) | ≤1 | ≥4 |
| Moxifloxacin (4^th^ generation) | ≤2 | ≥8 |
| Gatifloxacin (4^th^ generation) | ≤2 | ≥8 |
| Carbapenem |  |  |
| Imipenem | ≤2 | ≥8 |
| Lipopeptide |  |  |
| Polymyxin B | ≤2 | ≥4 |

*Tazobactam is tested at a fixed concentration of 4 µg/ml.


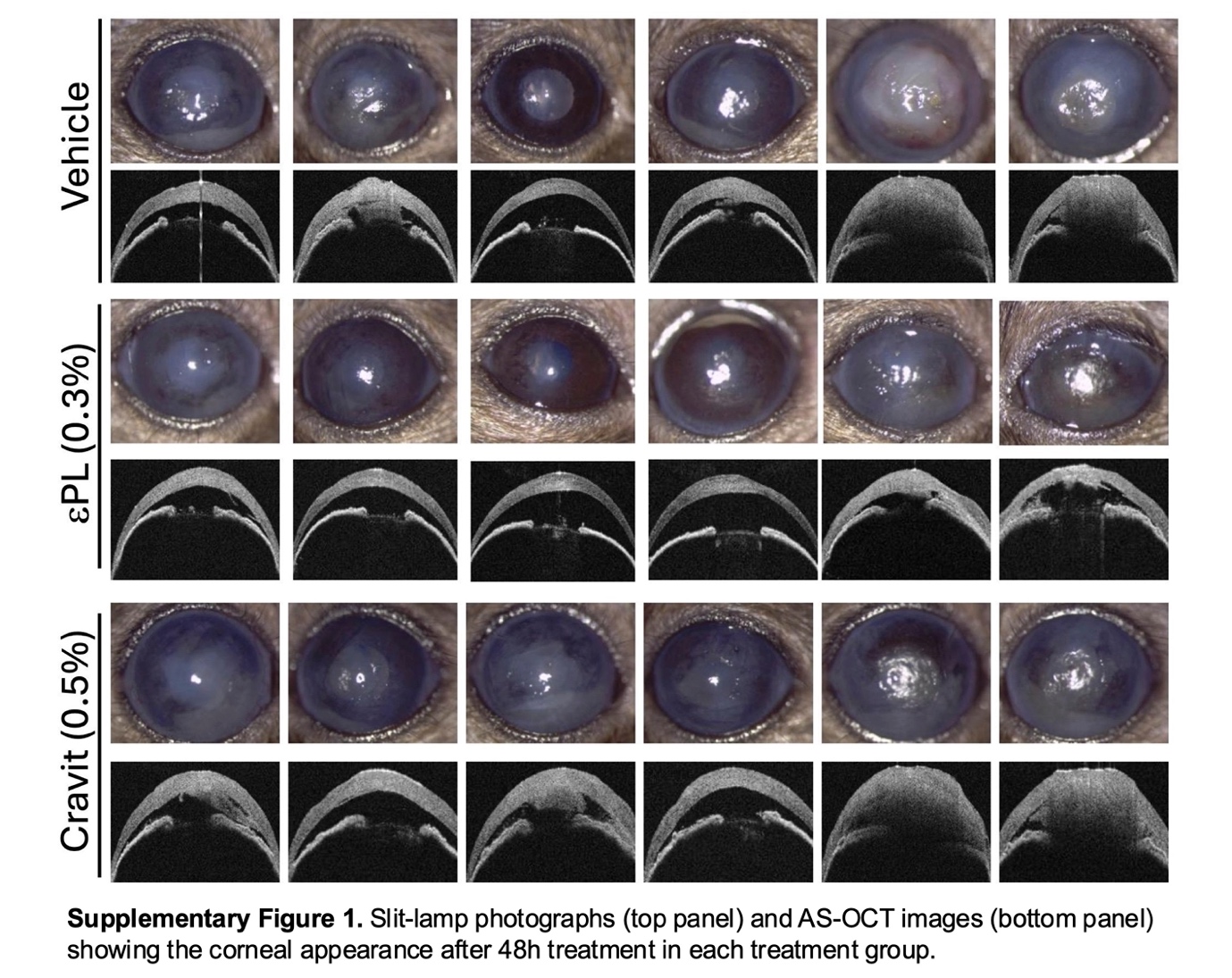

Supplement: Supplementary file 1 — Supplementary Table and Figure. [file 44259_2025_142_MOESM1_ESM.docx]
